# Exhausted CD8⁺ T cells promote ovarian cancer immunosuppression via CCL3-Driven M2 macrophage polarization

**DOI:** 10.1038/s41419-026-08772-4

**Published:** 2026-06-17

**Authors:** Yue Li, Jin Cheng, Fei Liu, Junjie Yi, Zhefeng Li, Yanheng Yu, Mei Jiang, Xiaoting Zhao, Wentao Yue

**Affiliations:** Central Laboratory, Beijing Obstetrics and Gynecology Hospital, Capital Medical University, Beijing Maternal and Child Health Care Hospital, Beijing, China

**Keywords:** Tumour immunology, Extracellular signalling molecules, Ovarian cancer, Cancer microenvironment, Tumour immunology

## Abstract

High-grade serous ovarian carcinoma (HGSOC) is an aggressive malignancy marked by high recurrence rates, poor prognosis, and limited response to immune checkpoint inhibitors, primarily attributable to its immunologically “cold” tumor microenvironment (TME). To profile the immunological landscape of HGSOC, we conducted single-cell RNA sequencing (scRNA-seq) on 84,065 cells from tumor tissues of eight treatment-naïve patients and one normal ovarian tissue, identifying six major cell clusters and revealing substantial immune cell infiltration. Further analysis of CD8⁺ T cells identified two key subpopulations—precursor and terminally exhausted T cells—and delineated their developmental trajectories. The accumulation of exhausted CD8⁺ T cells (Tex) suggested an immunosuppressive TME. Integrated trajectory inference and high-dimensional weighted gene co-expression network analysis (hdWGCNA) identified CCL3 as a novel hub gene specifically expressed in Tex cells. Communication analysis suggested that Tex cells may interact with M2 macrophages via the CCL3–CCR1 ligand–receptor axis. Functional validation confirmed that: (1) secretomes from Tex cells—but not effector T cells—significantly promoted M2 polarization in both THP-1 and bone marrow-derived macrophages (CD206⁺ THP-1: 83.8% vs. 51.4%, p < 0.001; CD206⁺ BMDM: 72.4% vs. 41.5%, p < 0.001); and (2) recombinant CCL3 acted synergistically with IL-4/IL-10 to further enhance M2 polarization (59.2% vs. 37.7%, p = 0.008). Collectively, our findings unveil a previously unrecognized immunoregulatory axis whereby exhausted CD8⁺ T cells drive immunosuppression via CCL3–CCR1–mediated communication with M2 macrophages, presenting a promising therapeutic target to reverse the immune-cold TME in HGSOC.

## Introduction

High-grade serous ovarian carcinoma (HGSOC), the most common and aggressive subtype of epithelial ovarian cancer, accounts for over 70% of ovarian cancer-related deaths [1, 2]. It is usually diagnosed at an advanced stage. Due to metastasis, recurrence, and chemoresistance, the five-year survival rate for patients is around 30%–40% [3]. Tumor immunotherapy, particularly immune checkpoint inhibitors (ICIs), has achieved revolutionary success in various malignancies, including melanoma and non-small cell lung cancer [4–6]. However, their efficacy in HGSOC remains limited, with objective response rates generally below 10%–15% [7]. This resistance is largely attributed to a highly immunosuppressive TME, which weakens or evades the body’s antitumor immune response [7–9].

The HGSOC TME is a complex ecosystem comprising diverse immune and stromal cells. T-cell exhaustion is a hallmark of this immunosuppressive ecosystem [10]. Exhausted T cells progressively lose their effector functions, including cytokine production and cytotoxic activity, under chronic stimulation from antigen-presenting cells or inflammatory signals. This is accompanied by the sustained overexpression of multiple co-inhibitory molecules (e.g., PD-1, TIM-3, and LAG-3) [11–13]. Extensive clinical and pathological studies demonstrate that the level of infiltrating exhausted T cells within HGSOC tissues is significantly associated with rapid disease progression, recurrence, and reduced overall patient survival. These functionally impaired T cells fail to eliminate tumor cells effectively and may also constitute a substantial barrier to ICI therapy [14]. However, the precise molecular mechanisms driving T-cell exhaustion and its crosstalk network in the HGSOC TME remain unclear.

In parallel with T cell dysfunction, tumor-associated macrophages (TAMs)—particularly those skewed toward the M2 phenotype—constitute another prominent feature of the HGSOC immune microenvironment. Increasing evidence suggests that these two immunosuppressive populations are not isolated but may interact synergistically to reinforce immune evasion [15, 16]. Understanding the potential crosstalk between exhausted T cells and M2 macrophages is thus critical for unraveling the full architecture of immune suppression in HGSOC.

Concurrently, macrophages—the most abundant immune cell population in the TME—polarize toward an M2 phenotype in HGSOC, exhibiting immunosuppressive and pro-tumor functions [17–19]. These M2 macrophages promote immune evasion and tumor progression through multiple mechanisms, including upregulating expression of metabolic enzymes such as arginase-1 (ARG-1) and indoleamine 2,3-dioxygenase (IDO), thereby depleting nutrients essential for T cell activation, and recruiting regulatory T cells (Tregs) to further suppress immune responses [20]. Multiple clinical studies confirm that high-density infiltration of M2 TAMs in tumor tissues is an independent risk factor for poor prognosis in HGSOC patients [21–24].

Traditionally, M2 TAMs were considered to be the ‘cause’, directly inducing and exacerbating T-cell exhaustion through these mechanisms and forming the cornerstone of the immunosuppressive microenvironment [15, 16, 25, 26]. However, the bidirectional nature of this interaction remains poorly defined. In this study, we reveal a previously unrecognized mechanism in which exhausted CD8⁺ T cells actively promote M2 macrophage polarization through CCL3 secretion. Our findings suggest that, within the HGSOC TME, exhausted T cells are not merely passive “victims” of immunosuppression but active “contributors” that reshape macrophage functional states. This discovery provides new insights into the complex intercellular dialogue driving immune escape in HGSOC.

## Results

### ScRNA-seq analysis of HGSOC and characterization of T-cell atlas

**Fig. 1:**
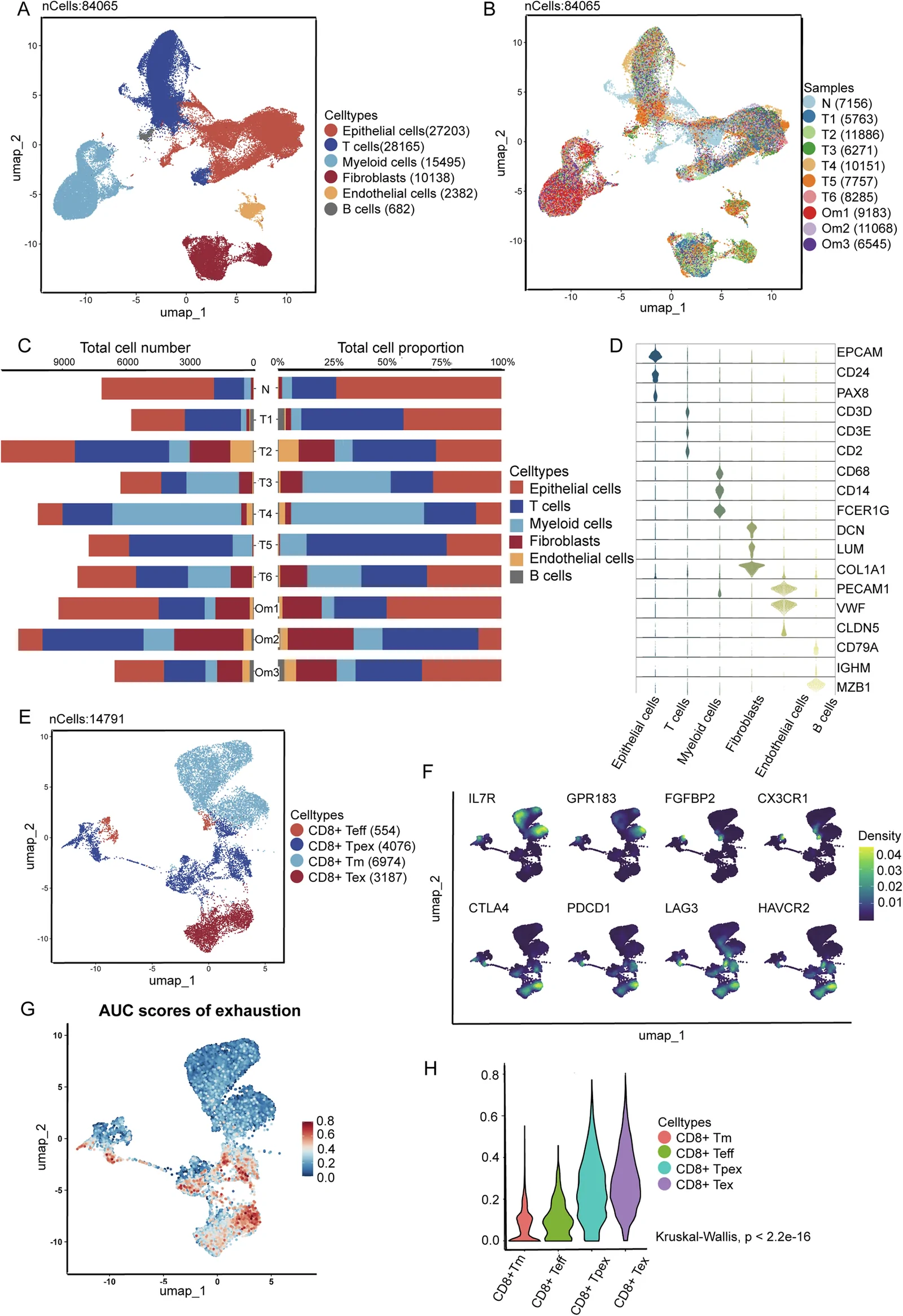
Single-cell landscape of T cells in HGSOC. **A,** **B** A single-cell atlas is displayed using a Uniform Manifold Approximation and Projection (UMAP) plot. This atlas comprises 84,065 cells from six annotated cell types. Epithelial cells and T cells account for the largest proportions. **C** Bar charts showing the cellular composition of each sample. These charts indicate a significant increase in T cells and myeloid cells in tumor tissue. N: Normal ovary. T: Primary tumor. Om: Omentum metastasis. **D** Violin plots showing the expression of classic markers used for cell type annotation across cell clusters, indicating the validity of our clustering annotation. **E** UMAP plot showing CD8^+^ T cells after further clustering and annotation based on their functional roles. In HGSOC, Tex and Tpex account for over half of the CD8^+^ T cells. **F** UMAP plot showing the expression of markers associated with different states of T lymphocytes across subpopulations. **G** and **H** Feature and violin plots showing T cell exhaustion scores calculated by AUCell.

To investigate the immune landscape of HGSOC, we performed single-cell RNA sequencing (scRNA-seq) on nine cases of tumor tissue from eight HGSOC patients, along with one normal ovarian tissue from one patient with uterine fibroids. After quality control and filtering (Fig. S1A), a total of 84,065 cells were retained for downstream analysis. Following normalization, batch correction, dimensionality reduction, and clustering, the cells were categorized into 23 distinct clusters (Fig. S1B). Based on classical markers, we annotated six major cell types: epithelial cells (EPCAM, CD24, PAX8), T-lymphocytes (CD3D, CD3E, CD2), myeloid cells (CD68, CD14, FCER1G), fibroblasts (COL1A1, LUM, DCN), endothelial cells (PECAM1, VWF, CLDN5) and B-lymphocytes (CD79A, IGHM, MZB1) (Figs. 1A, 1B and 1D). Notably, both the absolute number and proportion of T cells and myeloid cells were significantly higher in tumor tissue compared to normal tissue (Fig. 1C). This finding suggests that HGSOC represents a functionally ‘cold’ tumor, in which immune cells are abundantly present but functionally impaired within an immunosuppressive TME. This apparent disconnect between immune cell abundance and function prompted us to further investigate the functional state of CD8⁺ T cells, the key cytotoxic effectors.

We therefore performed clustering, annotation, and subpopulation analysis of CD8^+^ T cells. Based on classic markers, CD8⁺ T cells were categorized into four subpopulations: Tm (memory T cells), Teff (effector T cells), Tpex (precursor exhausted T cells), and Tex (exhausted T cells) (Fig. 1E). Feature plots illustrated the expression of memory T cell markers (IL7R, GPR183), effector T cell markers (FGFBP2, CX3CR1) and exhausted T cell markers (CTLA4, PDCD1, LAG3, HAVCR2) in T cells (Fig. 1F). Among these subpopulations, we focused on Tpex and Tex cells because they represent critical stages along the T cell exhaustion trajectory. Tpex cells express inhibitory receptors while retaining the potential to differentiate into terminally exhausted T cells [27, 28], whereas Tex cells showed high expression of exhaustion markers, consistent with a terminally exhausted phenotype. To further validate the rigor and rationality of this subpopulation, the exhaustion score for each CD8^+^ T cell was calculated using the AUCell R package and visualized in a feature plot and violin plot (Fig. 1G, H). These findings highlight Tpex and Tex as key players in the immune suppression observed in HGSOC, warranting further investigation into their differentiation trajectories and potential roles in shaping the TME.

### Revealing the exhaustion trajectory of CD8^+^ T cells

**Fig. 2:**
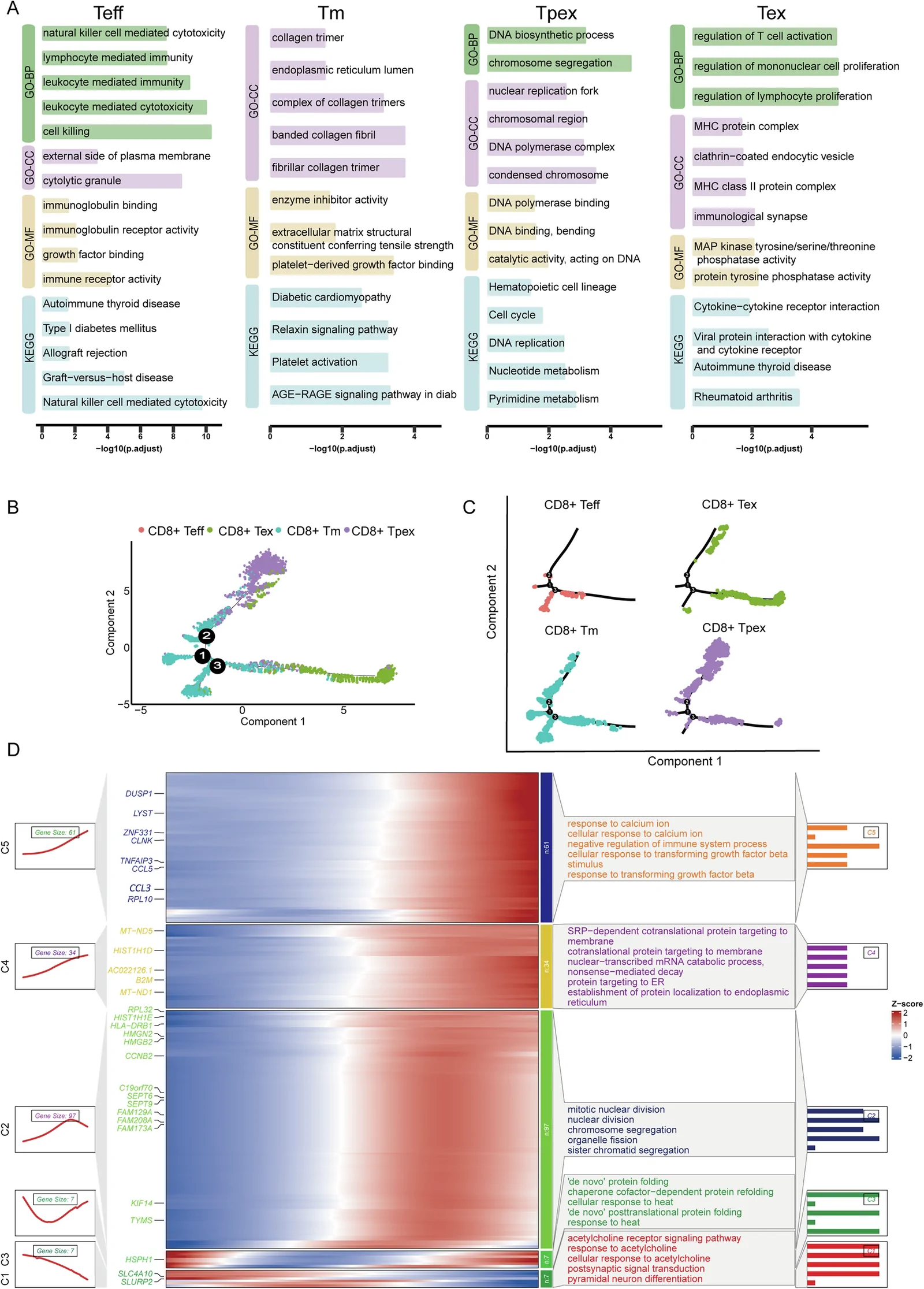
Pseudotime analysis of T lymphocytes in scRNA sequencing. **A** GO and KEGG enrichment analyses were performed on each T cell subset. Bar length represents the magnitude of the p-value. **B** Overall trajectory analysis of CD8^+^ T cell subgroups. **C** Hierarchical trajectory plot outlining the subgroups of CD8^+^ T cells. **D** Heatmap showing the expression dynamics of pseudotime-dependent genes, organized into five distinct modules (C1–C5). The bar charts on the right indicate the enriched pathways associated with each module.

Next, we performed KEGG and GO enrichment analyses on each T cell subset. While Teff and Tm cells showed enrichment in expected cytotoxic and tissue-interaction pathways [29], the functional enrichment of Tpex was concentrated in proliferation-related pathways, such as the cell cycle and DNA replication. This is consistent with previous studies that have suggested that precursor-exhausted T cells have the capacity to continuously replenish exhausted T cells [27, 28]. Tex cells were associated with weakened MAPK signaling and enhanced inhibitory phosphatase activity, collectively leading to inefficient TCR signal transduction, which is a hallmark feature of exhaustion. (Fig. 2A)

To further characterize the trajectory of T cell progression to exhaustion and identify the link between Tpex and Tex, we performed a pseudotime trajectory analysis of the annotated CD8^+^ T cells using the R package Monocle (v2.28.0). Pseudotime analysis revealed that memory T cells and effector T cells occupied the earliest positions along the pseudotime axis, while precursor exhausted T cells and exhausted T cells were located at the ends of the two trajectories (Fig. 2B, C). These results depict the developmental trajectory of T cells towards an exhausted state, suggesting that precursor exhausted T cells and terminal exhausted T cells represent distinct differentiation pathways rather than a linear progression.

To delineate the transcriptional dynamics associated with CD8⁺ T cell exhaustion along the pseudotime trajectory, we identified gene sets exhibiting significant expression changes. These genes were clustered into five distinct patterns using the R package ClusterGVis. While modules C1 to C3 displayed transient or biphasic expression trends—either declining (C1), rising then slightly decreasing (C2), or dipping then recovering (C3)—the C4 and C5 modules exhibited a consistent upregulation throughout the process of T cell exhaustion (Fig. 2D), highlighting their potential role in driving functional exhaustion.

We therefore focused on the C4 and C5 modules, which reflect monotonic activation during exhaustion progression. Enrichment analysis revealed that C4 is primarily involved in protein targeting, whereas C5 is significantly associated with negative regulation of immune system processes, response to calcium ions, and response to TGF-β. Given their sustained upregulation during exhaustion, we propose C4 and C5 as key candidate regulators of T cell dysfunction.

### Identification of hub genes involved in CD8^+^ T cell exhaustion

**Fig. 3:**
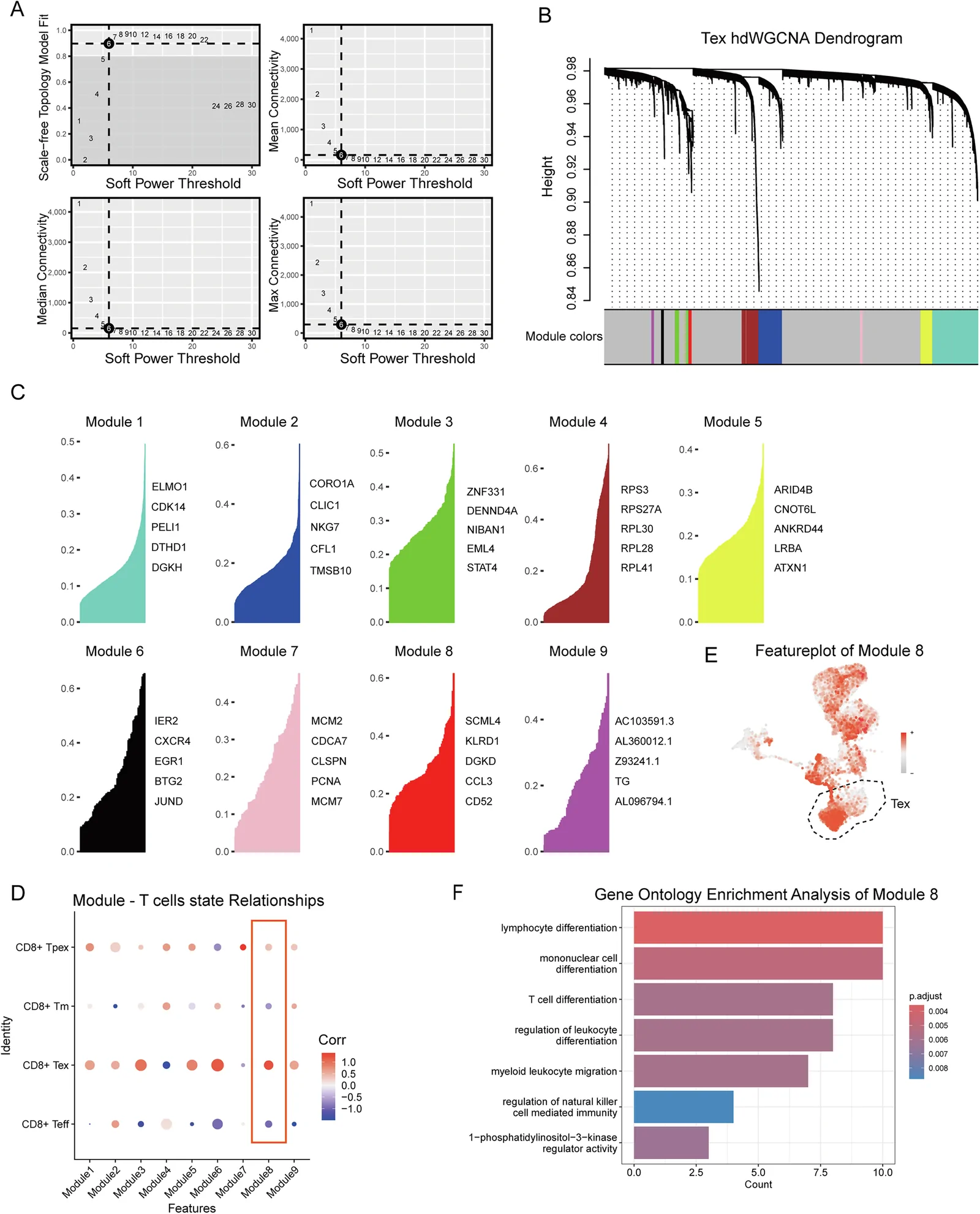
Identification of candidate T cell exhaustion-related genes. **A**, **B** Weighted gene co-expression network analysis was constructed among T cells. (See Materials and methods section) **C** The first 5 genes of each module, ranked by eigengene-based connectivity (kME). **D** Dot plot of the correlation between the state of T lymphocytes and identified modules. Module 8 is the most relevant to T-cell exhaustion. **E** UMAPs colored by gene expressions in Module 8 of T lymphocytes. Module 8 was enriched in exhausted T cells. **F** Bar plot of the GO functional enrichment analysis of Module 8. Most pathways are enriched in pathways associated with lymphocyte differentiation.

To further identify hub genes within these modules and validate their network centrality, we applied high-dimensional weighted gene co-expression network analysis (hdWGCNA). Using a soft threshold of 6, a scale-free network of CD8^+^ T cell subsets was created. This yielded optimal connectivity and identified 9 gene modules in total (Figs. 3A and 3B). Module eigengenes (MEs) were computed, and the top 5 most representative genes per module were displayed to illustrate module expression patterns (Fig. 3C). To identify the key modules most relevant to T cell exhaustion, we calculated the correlation between MEs of each module and the different T cell states. We then presented these correlations in bubble plots. Of all the modules, only Module 8 was positively correlated with both exhaustion subpopulations and negatively correlated with normal functional T cells (Fig. 3D). The UMAP projection of Module 8 gene expression displayed a spatial distribution that overlapped notably with that of the previously calculated T-cell exhaustion scores (Fig. 3E). To gain a preliminary understanding of Module 8’s biological function, we performed a GO enrichment analysis. The results showed that Module 8 was enriched for immune cell differentiation and regulation, especially lymphocyte differentiation, thus further validating Module 8 (Fig. 3F).

### CCL3 Orchestrates CD8^+^ T Cell Exhaustion and Macrophage-Mediated Immunosuppression

**Fig. 4:**
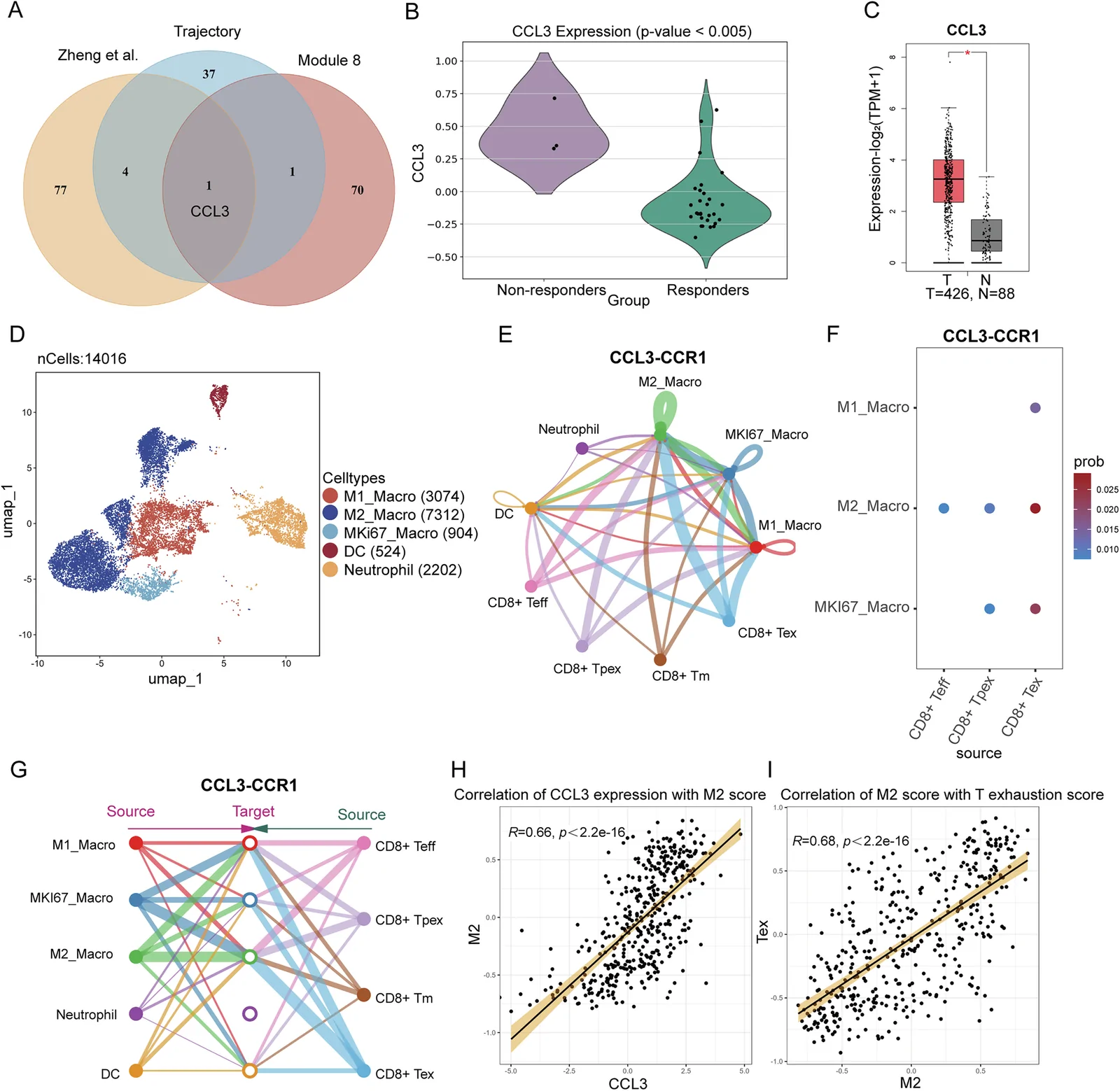
Exhaustion of T cells is associated with CCL3, and communicates most significantly with M2 macrophages. **A** Venn diagram demonstrated that three genes exhibited overlap among specific genes of pseudotime trajectory analysis and genes in Module 8, as well as exhausted CD8^+^ T cell genes identified by Zheng et al. CCL3 has been identified as the gene most closely associated with exhausted T cells. **B**, **C** CCL3 expression in TCGA datasets and GSE222556 correlates with chemotherapy response. **B** CCL3 is expressed at a higher level in tumors of chemotherapy non-responders than in those of responders. (n (non-responders vs. responders) = 3 vs. 27, p < 0.005) **C** CCL3 is expressed at higher levels in tumor tissues (T) than in normal tissues (N). (n (T vs. N) = 426 vs. 88, *p < 0.05, **p < 0.01, ***p < 0.001, ****p < 0.0001 in t-test.) **D** UMAP of myeloid cells in scRNA-seq data shows that macrophages account for the largest proportion of these cells. Among them, M2 macrophages are the most common type. **E,** **F**, and **G**. Network plot, dot plot, and hierarchy plot show the interactions of CCL3-CCR1 L-R pairs between myeloid cells and T lymphocytes. Intercellular communication suggests that exhausted T cells interact primarily with M2 macrophages via the CCL3-CCR1 axis. **H** Demonstration of the correlation between CCL3 expression and gene sets of M2 macrophages. **I** Demonstration of the correlation between gene sets of M2 macrophages and exhausted T lymphocytes.

To identify key genes of T-cell exhaustion, we integrated differentially expressed genes from pseudotime trajectory modules (C4/C5 module), top 10 weighted genes from module 8 of hdWGCNA, and published exhaustion signature genes (Zheng et al.). Among overlapping candidates, CCL3 (also known as MIP-1α) emerged as a central hub gene (Fig. 4A). Validation in clinical cohorts revealed that CCL3 expression was significantly elevated in both chemotherapy non-responders (GSE222556 dataset; Fig. 4B) and ovarian cancer tissues (TCGA database; Fig. 4C), suggesting its clinical relevance in HGSOC immune evasion.

To investigate its potential immunoregulatory role, we conducted intercellular communication analysis. Our results showed that exhausted T cells primarily communicate with macrophages (Fig. S2). Next, we focused our attention on macrophage subpopulations. Myeloid cells were re-clustered into five subsets: M1 macrophages (M1_Macro), M2 macrophages (M2_Macro), proliferative macrophages (Mki67_Macro), dendritic cells (DC), and neutrophils (Neutrophils) (Fig. 4D). CellChat analysis revealed that Tex cells exhibited heightened cell-cell communication, with M2 macrophages as primary recipients (Fig. 4E). Focusing on ligand-receptor pairs, we found that Tex-derived CCL3 signals were predominantly received by M2 macrophages via CCR1 (Fig. 4F, G).

These findings suggest that Tex-secreted CCL3 promotes immunosuppression and tumor progression in HGSOC by activating M2 macrophages. Supporting this, CCL3 expression strongly correlated with M2-associated gene signatures (Fig. 4H), while exhausted T-cell signatures showed a positive correlation with M2 signatures (Fig. 4I).

### Functional validation of CCL3-mediated M2 macrophage polarization by exhausted T cells

**Fig. 5:**
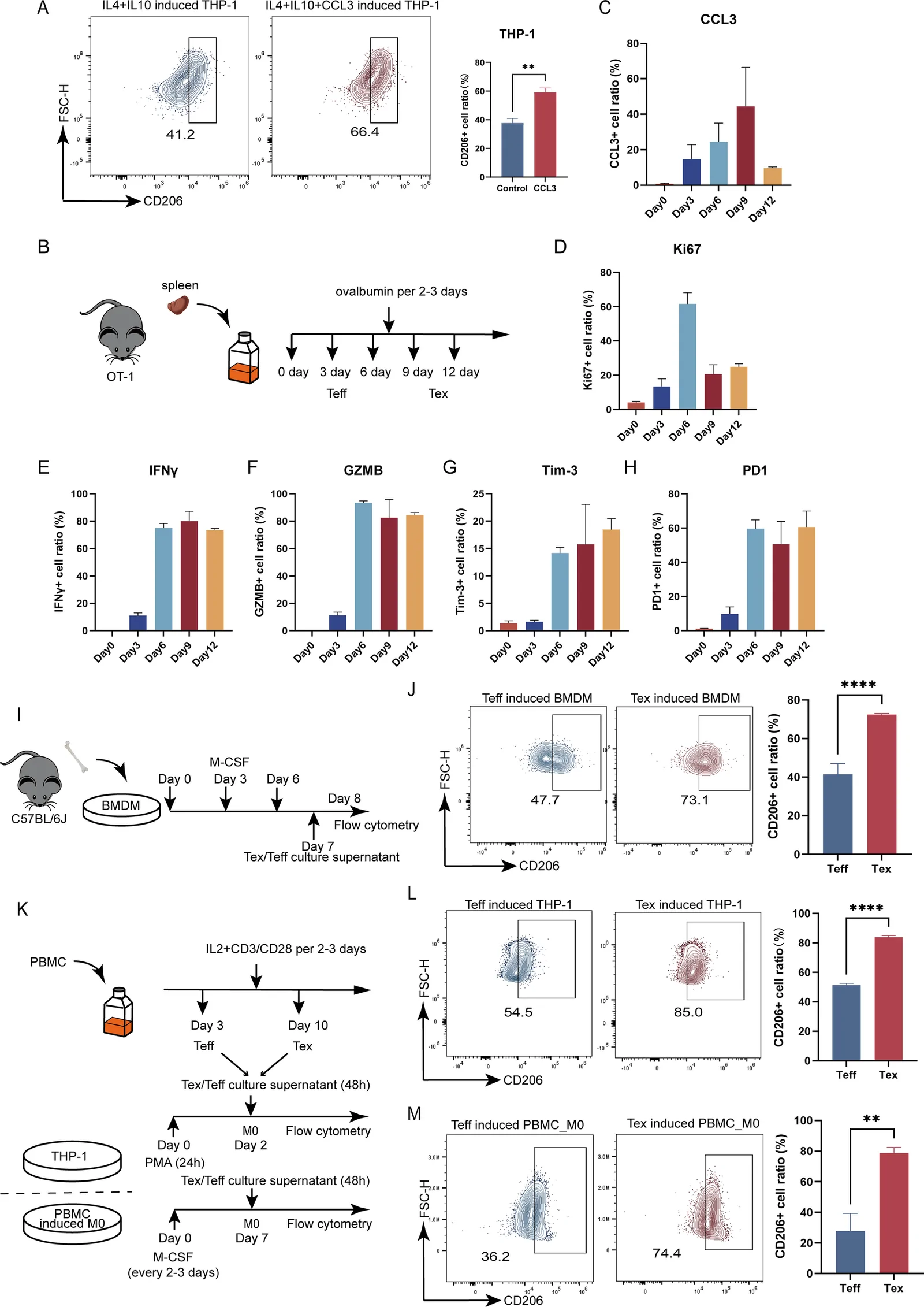
The secretion of CCL3 by exhausted T cells is a key driver of M2 macrophage polarization. **A** Flow cytometry analysis of THP-1 treated with IL4, IL10 (as control group), and IL4, IL10, CCL3 (as CCL3 group), respectively. (n = 5, Control vs. CCL3: 37.7% ± 3.2% vs. 59.2% ± 2.9%, p = 0.008) **B** Splenocytes from OT-1 mice were sorted for CD8^+^ T cells and stimulated to activation and exhaustion states by adding ovalbumin (10 ng/mL). **C-H** Bar plots showing CCL3 levels, proliferation levels, exhaustion status, and T cell killing capacity over time of stimulation, as detected by flow cytometry. **I** The flowchart demonstrates the use of Tex and Teff supernatants to incubate BMDM-induced polarization. **J** Flow cytometry analysis demonstrating the proportion of CD206-positive BMDM induced by Teff and Tex supernatant. (n = 6; Tex vs. Teff: 72.4% ± 0.6% vs. 41.5% ± 5.6%, p < 0.001) **K** Schematic illustration of the human cell co-culture system. CD8⁺ T cells isolated from PBMCs were stimulated with CD3/CD28 antibodies and IL-2 to generate effector T cells (Teff, day 3) and exhausted T cells (Tex, day 10). Conditioned media from Teff and Tex cells were then applied to PMA-differentiated THP-1 macrophages. **L** Flow cytometric analysis of CD206 expression in THP-1 macrophages following 48-hour incubation with Teff or Tex conditioned media (n = 3). **M** Flow cytometric analysis of CD206 expression in PBMC-derived primary human macrophages after 48-hour incubation with conditioned media from Teff or Tex cells (n = 4). Data are presented as mean ± SD. (*p < 0.05, **p < 0.01, ***p < 0.001, ****p < 0.0001 in t-test).

We tested the hypothesis that exhausted CD8⁺ T cells promote M2 polarization of macrophages through CCL3 secretion, as predicted by our bioinformatics analysis. First, we differentiated THP-1 cells into macrophages with PMA (phorbol myristate acetate, 100 ng/mL) treatment and divided them into two groups: a control group and a CCL3-treated group. The control group was treated with IL-4 (10 ng/mL) and IL-10 (10 ng/mL) for 24 hours. The CCL3 group received an additional treatment of recombinant CCL3 protein (50 ng/mL) along with the aforementioned cytokines for the same 24 hours. After flow cytometry analysis, we found that the percentage of CD206-positive macrophages was higher in the CCL3-treated group (n = 5, 59.2% ± 2.9% vs. 37.7% ± 3.2%, p = 0.008) (Fig. 5A). Next, we examined CCL3 expression in effector and exhausted T cells. To establish an in vitro T cell exhaustion model, we first harvested spleens from OT-1 mice and sorted and cultured the CD8^+^ T cells using T Cell Expansion Medium containing Ova (ovalbumin, 10 ng/mL) (Fig. 5B). We used flow cytometry to test whether the exhaustion model was successfully constructed by detecting the proliferative capacity (Ki67) (Fig. 5D), the killing capacity (GZMB and IFN-γ) (Figs. 5E and 5F), the exhaustion state (PD-1 and Tim3) (Figs. 5G and 5H), and the amount of CCL3 at 0, 3, 6, 9, and 12 days (Fig. 5C) for T cells, respectively. The results showed that, as stimulation time increased, T cell proliferation increased in the early stage and decreased in the late stage. T cell killing capacity significantly increased in the early stage and decreased slightly in the late stage. Exhaustion markers were progressively upregulated. These results are consistent with the pattern of T cell exhaustion. Based on these findings, we discovered that CCL3 expression was significantly elevated in late-exhausted T cells on day 9. Using this model, we designated CD8^+^ T cells harvested from day 3 cultures as effector T cells (Teff) and those harvested from day 9 cultures as exhausted T cells (Tex).

Next, we extracted bone marrow-derived macrophages (BMDM) from the femurs of mice. We flushed out the bone marrow with PBS, and we filtered the suspension through a 70-μm cell strainer. Erythrocytes were lysed and resuspended in 1640 medium containing M-CSF (macrophage colony-stimulating factor) at a concentration of 25 ng/mL (Fig. 5I). The BMDM cultured for seven days were divided into two groups and incubated with Teff and Tex culture supernatants for 24 hours. These groups were called the Teff group and the Tex group, respectively. Flow cytometry revealed that the percentage of CD206-positive BMDMs cultured with Tex supernatant was significantly higher than that of the Teff group (n = 6; 72.4% ± 0.6% vs. 41.5% ± 5.6%, p < 0.001) (Fig. 5J).

To validate these findings in human cells, we employed two complementary systems. In the first model, CD8^+^ T cells isolated from human peripheral blood mononuclear cells (PBMCs) were stimulated with IL-2 (20 ng/mL) and CD3/CD28 antibodies; day-3 cells served as Teff and day-10 cells as Tex. Conditioned media from these cells were applied to PMA-differentiated THP-1 macrophages for 48 hours (Fig. 5K). The Tex group showed substantially increased CD206+ cell frequencies compared to the Teff group (n = 3; 83.8% ± 1.2% vs. 51.4% ± 1.2%, p < 0.001; Fig. 5L). In the second model, to further recapitulate physiological conditions, we generated primary human macrophages by differentiating PBMC-derived monocytes with M-CSF (50 ng/mL) for 7 days, followed by 48-hour exposure to Teff or Tex conditioned media. Consistent with our previous observations, Tex supernatants induced significantly greater M2 polarization than Teff supernatants (n = 4; 78.9% ± 3.5% vs. 27.8% ± 11.5%, p = 0.002; Fig. 5M). Together, these findings demonstrate across murine and human systems, including both immortalized cell lines and primary cells, that exhausted CD8⁺ T cells functionally promote M2 macrophage polarization through CCL3 secretion, supporting a novel immunosuppressive axis that may contribute to HGSOC progression.

## Discussion

Exhausted T cells are a central component of the TME and play a key role in shaping its immune evolution. Within the TME, these cells represent the outcome of continuous immune pressure, maintaining antigen recognition but showing reduced effector function. Through their altered cytokine production and high expression of inhibitory receptors, exhausted T cells affect the balance between immune activation and suppression, contributing to tumor persistence and immune escape [30, 31]. In our study, we found that exhausted T cells are not only a result of immune evolution but also active participants that strengthen the immunosuppressive environment, further promoting tumor–immune coevolution and resistance to immunotherapy.

Recent research by Hor et al. (Nature 2025) provides important context for understanding the functional heterogeneity of exhausted T cells [32]. Their study demonstrated that PD-1 signaling in tumor-draining lymph nodes serves not merely as an inhibitory checkpoint, but as a critical regulator that fine-tunes TCR signals to maintain the stemness and selective expansion of high-affinity stem-like T cells (TSL). Mechanistically, strong TCR engagement by high-affinity clones drives PD-1 upregulation, which in turn attenuates signal intensity and prevents premature terminal differentiation, thereby forming a negative-feedback loop that preserves the regenerative capacity of the anti-tumor T cell pool. This finding, demonstrating that a classically “inhibitory” molecule can exert context-dependent protective functions, resonates with our observations regarding CCL3. Importantly, their work also highlights a fundamental distinction between exhaustion stages: while early-stage TSL cells can be protected by PD-1-mediated signal attenuation and serve as a renewable reserve for sustained anti-tumor responses, T cells that progress into terminal exhaustion under chronic antigenic stimulation may acquire distinct functional properties that extend beyond simple loss of effector function. Our findings extend this conceptual framework by demonstrating that, within the HGSOC microenvironment, terminally exhausted CD8⁺ T cells are not merely dysfunctional bystanders but active participants that promote immunosuppression through CCL3-mediated M2 macrophage polarization. This observation suggests that the biological consequences of T cell exhaustion are stage-dependent, with terminal exhaustion potentially representing a functional transition toward active immunomodulation rather than passive dysfunction.

The chemokine CCL3 has traditionally been recognized as a classic pro-inflammatory factor, instrumental in recruiting and activating T cells during infection or vaccine responses [33–37]. However, our study uncovers a previously unrecognized immunosuppressive signaling axis in HGSOC, which challenges this conventional view. This finding reveals that CCL3 undergoes a functional reprogramming in the TME—transforming from an immune-stimulating factor into a tumor-promoting, immunosuppressive molecule. These findings suggest that CCL3 plays dual roles in immune regulation, depending on the immunological context. During acute infection or certain phases of anti-tumor immunity, it likely acts as an alarmin, promoting inflammatory responses. In stark contrast, within the chronic, immunosuppressive niche of HGSOC, CCL3 is hijacked to establish an immunosuppressive barrier that supports tumor growth and immune evasion. This context-dependent functional plasticity underscores the intricacy of immune regulation in cancer and implies that the function of immune factors must be considered in the broader context of the immune system. Our study significantly enriches the understanding of CCL3 in the immune evolution of HGSOC, establishing it not only as a traditional immune mediator but also as a critical effector in Tex-driven immunosuppression. These findings carry important clinical implications. They posit that CCL3 is not a unidirectional modulator but rather a context-dependent chemokine. Similar to CCL3, several immune mediators, including CCL2, CCL5, IL-6, IFN-γ and TGF-β, exhibit context-dependent bidirectional functions [38–42]. Depending on the cellular and microenvironmental context, they can act as either pro- or anti-tumor factors. This functional duality likely arises from differences in receptor expression and signaling intensity, as well as microenvironmental cues that dynamically reprogram their downstream pathways within the TME [43]. Therefore, understanding the precise molecular cues that govern the switch between the pro- and anti-tumor roles of CCL3 could unveil novel targets for future immunomodulatory therapies, allowing us to strategically disrupt its tumor-promoting functions while preserving its beneficial immune activities.

Our analysis revealed that elevated CCL3 expression and activation of the CCL3–CCR1 axis are strongly associated with resistance to platinum-based chemotherapy. CCL3–CCR1 signaling may activate the PI3K–Akt, NF-κB, and MAPK pathways, thereby enhancing cell survival, DNA damage repair, and anti-apoptotic programs in tumor or stromal cells [44–46]. Concurrently, CCL3-driven remodeling of the TME, through the recruitment and polarization of macrophages and regulatory T cells, may suppress chemotherapy-induced immunogenic cell death and foster a tolerogenic niche. Considering the pivotal role of M2 macrophages in drug resistance and immune evasion, the Tex–CCL3–M2 circuit may be a critical link between chronic T-cell dysfunction and therapeutic failure. Furthermore, since immune checkpoint blockade depends on functional effector T cells [47]. the accumulation of exhausted T cells and their M2-polarizing chemokine signals may predict poor responsiveness to immunotherapy [48]. Therefore, targeting the CCL3–CCR1 axis holds promise as a strategy to overcome chemoresistance and immune escape.

This study has several limitations. First, the small sample size (n = 8) limits the generalizability of our findings and requires validation in larger groups. Second, while we showed that CCL3 promotes M2 macrophage polarization, the downstream signaling pathways following CCR1 activation are still unknown, leaving the precise molecular mechanisms unclear. Third, the triggers that prompt exhausted T cells to secrete CCL3, as well as the regulatory mechanisms that govern CCL3’s dual functions within the TME, remain unknown. Future studies integrating multi-omics profiling and mechanistic validation are necessary to elucidate the complete CCL3–CCR1 signaling network and its role in immune modulation and therapeutic resistance.

In conclusion, our study reveals a previously unrecognized tumor-promoting role of CCL3 in the HGSOC immune microenvironment: CCL3 secreted by exhausted CD8⁺ T cells induces M2 polarization of macrophages, forming a positive feedback signaling axis of immunosuppression. This discovery underscores the dual nature of immune mediators—their function is not only molecule-dependent but also context-dependent. By dissecting the Tex–CCL3–M2 immunological axis, we offer a new mechanistic perspective on how chronic T cell dysfunction expands immunosuppression through myeloid cells. Given the druggable nature of this axis, targeting CCL3 or its receptor CCR1 may become a promising strategy for combining immunotherapy with resistance reversal, potentially bringing new clinical benefits to patients with HGSOC.

## Methods

### Human tissue specimens

The samples of the eight HGSOC cases and one normal ovarian tissue for scRNA-seq were obtained from patients attending the Beijing Obstetrics and Gynecology Hospital, Capital Medical University. Patients were enrolled after signing an informed consent form. The study was carried out according to the ethical guidelines of Beijing Obstetrics and Gynecology Hospital and approved by the hospital’s Ethics Committee (approval No. 2023-KY-080-01).

### Single-cell RNA sequencing

Following the diagnosis of HGSOC by rapid pathology, tissue samples from eight HGSOC patients and one patient with uterine fibroids were dissociated into single-cell suspensions for cell counting and cell viability determination by a cell counter. The prepared single-cell suspensions were then combined with a mixture of gel beads containing barcode information and enzymes, and finally encapsulated by oil droplets in microfluidic “single cross” junctions to form GEMs (Gel Bead-In-EMulsions). Subsequently, the GEMs underwent a process of cell lysis and reverse transcription, and the 10x Barcode was ligated to the cDNA product. Following this, the GEMs were broken up, and the oil droplets were fragmented for PCR amplification using the cDNA as a template. The construction of sequencing libraries was preceded by the implementation of a quality control procedure on the amplified products. The subsequent analysis of the data was performed using the Illumina NovaSeq sequencing platform.

### Quality control and data integration

The sequence data were used to map the reads to the human genome (GRCh38) employing Cellranger 7.0.0 (http://10xgenomics.com). The data were analyzed using the Seurat package (v4.3.0). The Seurat objects were initially created using the Read10X function, with the data from each of the nine samples undergoing quality control. Subsequently, the data were log-normalized, and homogenization and principal component analysis were performed after identifying highly variable genes. The RunHarmony function from the harmony package (v1.1.0) [49] was employed to eliminate batch effects. Subsequently, the number of principal components was determined, and UMAP downscaling was performed. Subpopulations were categorized and annotated based on markers characteristic of distinct cell types. The annotation process was facilitated by the SingleR package (v2.0.0) [50].

### Pseudotime analysis

We used the Monocle2 R package (v2.28.0) [51] for pseudotime analysis to reconstruct cell trajectories. Briefly, gene expression matrices were converted to Monocle-compatible CellDataSet objects. The DDRTree algorithm was used to reduce the dimensionality and construct the trajectory graph. The root node of the trajectory was set manually based on the biological significance of cell subpopulations. Cells were then sorted along the trajectory, and a pseudo-time value was assigned to each cell. We performed enrichment analysis and visualization of pseudotime-dependent genes using the ClusterGVis R package (v0.1.4).

### Cell–cell communication analysis

We used the CellChat R package (v2.1.0) [52] to analyze communication between two cell types: T cells and myeloid cells. First, we constructed CellChat objects and then used CellChatDB V2 as a ligand database. After filtering out populations of less than 10 cells, we inferred the intercellular communication network.

### hdWGCNA analysis

HdWGCNA identifies modules of highly co-expressed genes and provides context for these modules via statistical testing and biological knowledge sources [53]. With the scale-free topology model fit threshold set at > 0.8, a soft threshold of 6 was selected for the best connectivity. Correlations between modules and T-cell status were evaluated by the Spearman test.

### Statistical analysis

Statistical analyses were performed using R (v4.4.2), and GraphPad Prism (v9.0). For data meeting the assumptions of normality and equal variance, a two-tailed Student’s t-test was used for two-group comparisons, and one-way ANOVA followed by Tukey’s post-hoc test was applied for multiple groups. Survival data were analyzed using the log-rank test. All experiments were repeated at least three times. Sample sizes were determined based on previous literature and preliminary experiments to ensure adequate statistical power. For in vitro experiments, samples were not randomly allocated, and investigators were not blinded to the group allocations; however, all data analysis was performed using standardized automated software to ensure objectivity. Data are expressed as the mean ± standard deviation (SD). A p-value of less than 0.05 was considered statistically significant for all statistical comparisons.

### Cell culture

The human monocytic cell line THP-1 was purchased from BeNa Culture Collection (BNCC, Cat: BNCC358410). Cells were cultured in RPMI-1640 medium supplemented with 10% FBS and 1% penicillin-streptomycin. The identity of the THP-1 cell line was authenticated by Short Tandem Repeat (STR) profiling using a 21-STR amplification scheme (BeNa Culture Collection). The STR genomic profile of the cells was compared with reference databases including ATCC, DSMZ, JCRB, and ExPASy, showing a high match score (EV = 0.96) with the standard THP-1 profile (CVCL_0006) and no detectable multi-allele contamination. All cells were routinely tested for mycoplasma and remained negative throughout the experimental period.

### Modeling of T cell exhaustion

#### Mouse

Spleens were isolated from OT-1 mice and ground under a clean bench. The tissue homogenate was filtered using a 200 μm nylon filter and subjected to erythrocyte lysis. Next, Naïve CD8^+^ T cells were isolated using Naïve CD8a^+^ T Cell Isolation Kit (Cat: 130-104-075, Miltenyi Biotec). Finally, the isolated cells were cultured using ImmunoCult™-XF T Cell Expansion Medium (Cat: #10981, STEMCELL) and stimulated with 10 ng/mL ovalbumin (Cat: 763606, Biolegend). Cells were counted and replaced to maintain optimal cell density with fresh stimulation-supplemented medium every three days.

#### Human

CD8^+^ T cells were isolated from PBMCs (Cat: 130-096-495, Miltenyi Biotec) and stimulated using IL2 (Cat: HY-P7037B, MCE) and CD3/CD28 (Cat: #100-0784, STEMCELL) to construct T cell activation models and exhaustion models. Stimuli were replenished every 3-4 days.

### Isolation and differentiation of primary macrophages

#### Murine bone marrow-derived macrophages (BMDMs)

First, female mice (6–8 weeks old) were euthanized and sterilized in 75% alcohol for 10 min. Subsequently, the BM in the femur and tibia was flushed out and treated with red blood cell lysis buffer (Cat: R1010, Solarbio). BM cells were then cultured in 90% RPMI1640, 10% FBS medium with macrophage colony-stimulating factor (MCSF, Cat: HY-P7085A, MCE), and 1% streptomycin/penicillin at a density of 3×10^6^ per 6-well cell culture plate for 7 days, and the medium was changed every 2–3 days. After 7 days of induction, the obtained BMDMs were used for subsequent experiments.

#### Human PBMC-derived macrophages

Human peripheral blood mononuclear cells (PBMCs) were purchased from CTCC (Cat: CTCC-009-115, MEISEN). According to the manufacturer’s instructions, PBMCs were cultured in RPMI 1640 medium supplemented with 10% FBS. To induce macrophage differentiation, PBMCs were seeded and allowed to adhere for 4 hours, after which non-adherent cells were removed. The adherent monocytes were then cultured in complete medium supplemented with 50 ng/mL recombinant human M-CSF (Cat: HY-P73827, MCE) for 7 days. Successful differentiation into M0 macrophages was confirmed by morphological assessment and the ability to adhere to the culture surface.

### Flow cytometry

The cells to be tested were collected, and then, after centrifugation at 500 g for five minutes, the cells were resuspended in staining buffer (RPMI 1640 + 2% FBS medium) and labeled with surface antibodies. Then, the cells were fixed with Cytofix Fixation Buffer (Cat: 554655, BD), permeabilized with Perm/Wash Buffer (Cat: 554723, BD), and labeled with intracellular antibodies. Online analysis was then performed. The following antibodies were used: PD-1 (Cat: 135219, BioLegend), Tim3 (Cat: 119725, BioLegend), Ki67 (Cat: 563757, BD), CCL3 (Cat: IC450A, Bio-Techne), IFN-γ (Cat: 505841, BioLegend), GZMB (Cat: 372230, BioLegend), CD206 (Cat: 321105, BioLegend), CD86 (Cat: 305411, BioLegend), CD11b (Cat: 301329, BioLegend).

## Supplementary information

Supplementary figures

checklist

## Data Availability

Data have been deposited in the OMIX/NGDC database under accession PRJCA050388.
