# Supplementary material for: Exhausted CD8⁺ T cells promote ovarian cancer immunosuppression via CCL3-Driven M2 macrophage polarization
Source: Cell Death Dis. 2026 Jun 17;17(1):757. doi: 10.1038/s41419-026-08772-4 (PMC13518876; doi:10.1038/s41419-026-08772-4)
Supplement: Supplementary file 2 [file 41419_2026_8772_MOESM1_ESM.docx]

**Fig. S1.** A. Violin plots depicting key metrics of single-cell RNA sequencing data across different cell types/clusters. The left panel illustrates the distributions before filtering, while the right panel shows the distributions after filtering. The plots show the distribution of nFeature (number of detected genes per cell), nCount (total UMI counts per cell), mitochondrial ratio (proportion of mitochondrial gene UMI counts), ribosomal ratio (proportion of ribosomal gene UMI counts), and erythrocyte ratio (proportion of erythrocyte gene UMI counts). B. UMAP plot showing the results of dimensionality reduction and clustering analysis of single-cell RNA sequencing data. The plot illustrates the distribution of cells across 15 distinct clusters.

**Fig. S2.** Cell communication analysis using CellChat, depicting interactions between T cell subsets and other cell subsets. The left panel illustrates the number of interactions, showing the frequency of communication events between different cell types. The right panel displays the strength of interactions, highlighting the intensity of signaling pathways between the cell subsets.
